# Mitochondrial Bioenergetics and Quality Control Mechanisms in Health and Disease

**DOI:** 10.1155/2019/5406751

**Published:** 2019-01-20

**Authors:** Julio C. B. Ferreira, Marcelo A. Mori, Eric R. Gross

**Affiliations:** ^1^Department of Anatomy, Institute of Biomedical Sciences, University of Sao Paulo, SP, Brazil; ^2^School of Medicine, University of Sao Paulo, SP, Brazil; ^3^Department of Biochemistry and Tissue Biology, Institute of Biology, University of Campinas (Unicamp), SP, Brazil; ^4^Department of Anesthesiology, Stanford University School of Medicine, Stanford, California, USA

Since the first endosymbiotic event occurred, where a proteobacterium was engulfed by larger cells, evolutionary pressure was imposed into mitochondria in order to facilitate the higher energy output required for the evolution of complexity. As a result, mitochondria are also a primary source of reactive molecules involved in physiologic redox signaling and the cause of oxidative stress from pathological events [1]. Besides their central role in ensuring that energy demands are met, mitochondria also contribute to intercellular and intracellular processes such as controlling nuclear gene expression, ion homeostasis, and apoptosis [2]. Thus, maintaining mitochondrial integrity and functionality is critical for cellular homeostasis. As shown by the manuscripts assembled for this special issue, mechanisms for surveillance of mitochondria are complex and diverse. However, these manuscripts together echo the importance of assessing mitochondria for integrity and functionality to identify aberrant redox signaling or early signs of pathology [3]. In general, mitochondrial quality is controlled by a myriad of interconnected systems including (1) enzymatic and nonenzymatic elements capable of fighting oxygen-mediated mitochondrial toxicity [4], (2) mitochondrial proteases and chaperones responsible for the maintenance of mitochondrial proteostasis [5], and (3) a multilayer network of proteins involved in the control of mitochondrial morphology, location, and number [6]. Disruption of mitochondrial quality control mechanisms in general results in adverse effects that contribute to the establishment and progression of several diseases [7, 8]. Therefore, the development of pharmacological and nonpharmacological approaches capable of optimizing mitochondrial surveillance and quality control mechanisms is a promising tool to treat diseases [9, 10].

This special issue consists of 6 original articles and 2 review articles that broaden our understanding of the regulatory processes involved in mitochondrial bioenergetics, surveillance, and quality control mechanisms of mitochondria in health and disease. In turn, these manuscripts bring new insights into mitochondrial signaling mechanisms and for proposing novel approaches for diagnostics and therapeutics. They cover the topics of (1) redox signaling and oxidative stress, (2) mitochondrial proteostasis, (3) mitochondria-nucleus communication (mitochondrial retrograde signaling), (4) mitochondrial dynamics: mitochondrial biogenesis, fusion, and fission, (5) mitochondrial clearance (mitophagy), and (6) mitochondrial bioenergetics.

Two articles in this special issue focus on the interplay between mitochondria bioenergetics and oxidative stress in skeletal muscle physiology. M. Marrone et al. demonstrate that sarcopenia (i.e., age-related loss of skeletal muscle mass and function) is paralleled by impaired mitochondrial bioenergetics and excessive superoxide levels in both myoblasts and myotubes from skeletal muscle of elderly subjects. However, the direct impact of mitochondrial dysfunction on skeletal muscle regenerative capacity in ageing still needs to be addressed. M. Eimre et al. provide evidence that Wfs1-deficient mice, which display progressive loss of plasma insulin concentration, exhibit reduced mitochondrial oxygen consumption, with no changes in mass of the *soleus* skeletal muscle (abundant in slow twitch muscle fibers). In contrast, the *rectus femoris* skeletal muscle (abundant in fast twitch muscle fibers) has a significant reduction in mass along with increased mitochondrial content and improved bioenergetics. These differences in skeletal muscle metabolism in Wfs1-deficient mice should be further explored in order to understanding its possible contribution to diabetes or Wolfram syndrome.

Two other articles in this special issue focus on the role of mitochondrial metabolism in tumor biology. Many tumors are characterized by changes in the mitochondrial electron transport chain composition, which might have either positive or negative impact on energy production. R. G. Feichtinger et al. investigate the expression of oxidative phosphorylation complexes in human prostate carcinomas. There is an accumulation of oxidative phosphorylation complexes I, II, and V in carcinomas compared with benign tissue. Moreover, complex V levels (i.e., ATP5F1A subunit) have a positive correlation with early-onset prostate cancer. Whether these changes in complex abundance reflect functional alterations in mitochondria of tumor cells remains to be determined, although this is likely given the rate limiting characteristic of some of these complexes. In another article, G. H. Tamarindo et al. show that melatonin or docosahexaenoic acid supplementation reduces proliferation of PNT1A cancer cell line. Of interest, melatonin and docosahexaenoic acid have opposite effects on mitochondrial bioenergetics and hydrogen peroxide production in this cell line. Nonetheless, the contribution of mitochondrial biology to the impact of such interventions in PNT1A cancer cell line is still unclear.

Finally, two other articles in this special issue focus on the role of mitochondria as detoxifying systems capable of fighting aldehyde- and oxygen- mediated toxicity. L. G. M. Wijermars et al. demonstrates that impaired aldehyde metabolism, characterized by reduced activity of aldehyde dehydrogenase enzyme and accumulation of toxic reactive aldehydes, is associated with delayed graft function following kidney transplantation in humans. These findings suggest that measuring aldehyde metabolism can be a possible strategy to develop biomarkers to assess delayed graft function from a kidney transplant. In another article, R. J. Mailloux provides an updated view on the critical contribution of mitochondria to the degradation of intracellular hydrogen peroxide as well as its contribution to both redox signaling and oxidative stress. Considering that mitochondria have a high concentration of antioxidant defense enzymes, it is expected that therapies capable of changing mitochondrial content, number, and function will play a critical role in redox biology [1, 2, 6, 10]. In that sense, H.-Y. Lin et al. demonstrate that the reestablishment of mitochondrial network through overexpression of mitochondrial fusion-related proteins or downregulation of mitochondrial fission-related proteins reduces oxidative stress and improves insulin signaling in cybrid cells harboring diabetes mellitus-susceptible mtDNA haplogroup. Finally, S. Sivanesan et al. describe in a review article some possible connections between impaired energy metabolism and Alzheimer's disease.

We hope that this special issue provided new insights and fostered new ideas into mitochondrial biology and how it associates with health and disease.
